# Evaluating the impact of tai chi therapy on stress reduction among older adults

**DOI:** 10.6026/9732063002001877

**Published:** 2024-12-31

**Authors:** Siva Subramanian N., Mevada Zankhana Hareshbhai, Santhi S., Bhaskaran R., Jamuna Rani P., Mahalakshmi B.

**Affiliations:** 1 Department of Psychiatric Nursing, Nootan College of Nursing, Sankalchand Patel University, Visnagar, Gujarat - 384315, India; 2 Department of Psychiatric Nursing, Sri Ramachandra Faculty of Nursing, Sri Ramachandra Institute of Higher Education and Research(DU) Porur, Chennai - 600116, India; 3 Department of Psychiatric Nursing, Cherraan's college of Nursing, Coimbatore - 641014; 4 Department of Psychiatric Nursing, KMCH College of Nursing, Coimbatore, Tamil Nadu - 641048, India; 5 Department of Pediatric Nursing, Nootan College of Nursing, Sankalchand Patel University, Visnagar, Gujarat - 384315, India

**Keywords:** Tai Chi therapy, stress reduction, older adults, quasi-experimental, perceived stress scale, mehsana district

## Abstract

This study investigates the effectiveness of Tai Chi therapy in reducing stress among older adults in selected community areas of Mehsana District. A quasi-experimental pre-test and post-test design was used with 60 participants aged 60-80, divided into experimental and control groups. The experimental group practiced Tai Chi therapy under supervision for 14 days, while the control group received no intervention. Stress levels were measured using the Perceived Stress Scale (PSS) before and after the intervention. Results showed a significant reduction in stress in the experimental group, with the mean pre-test score of 27.2 dropping to 10.1 post-intervention. In contrast, the control group showed no significant change. The t-test results confirmed Tai Chi's effectiveness in reducing stress (t = 10.43, p < 0.05) for the experimental group, with no significant association between stress reduction and demographic variables. These findings support Tai Chi as a beneficial intervention for managing stress among older adults.

## Background:

Stress among older adults is a significant public health issue, with nearly 2 billion people expected to be over 60 by 2050 [1]. Aging brings physical and mental challenges, including chronic illness, reduced mobility and social isolation, which increase stress and impact overall health [2]. Chronic stress in older adults has been linked to cardiovascular issues, weakened immunity and mental health disorders like depression and dementia. Thus, effective stress management is crucial to improve the quality of life in this demographic [3]. In India, where 10% of the population is aged 60 or above, stress management among older adults is challenging, especially in rural areas where mental health services are limited [4]. Conventional methods, like medication and therapy, are not always accessible or feasible, prompting interest in low-cost, community-friendly alternatives. Tai Chi-a gentle, meditative form of martial arts combining slow movements, breath control and mindfulness-has shown promise as a feasible stress reduction intervention. Tai Chi's controlled, low-impact exercises are particularly suitable for older adults and have been associated with reduced anxiety, improved mood and enhanced physical functioning [5]. Research supports Tai Chi's effectiveness in stress management for older adults. Kuang *et al.* (2024) reviewed trials finding Tai Chi significantly lowered stress in older populations, [6] while Irwin *et al.* (2017) found that a 12-week Tai Chi program reduced anxiety and depression symptoms in seniors with chronic health issues [7]. In Indian rural communities, Tai Chi could offer an accessible, non-invasive solution for stress management. This study examines the impact of Tai Chi on stress reduction in older adults in Mehsana District, assessing its potential as an integrative, community-based intervention.

## Methodology:

## Research design:

A quasi-experimental, pre-test and post-test control group design was used to assess Tai Chi's effectiveness in reducing stress among older adults [8, 9].

## Setting:

The study took place in community areas of Mehsana District, supported by the Kharavada Primary Health Centre, which provided necessary infrastructure and participant access.

## Population and sample:

The target population consisted of older adults aged 60-80 with mild to moderate stress. A purposive sample of 60 participants was divided into experimental (n=30) and control (n=30) groups. Inclusion criteria included willingness to consent and physical ability to perform Tai Chi.

## Variables:

[1] Independent Variable: Tai Chi therapy

[2] Dependent Variable: Stress levels, measured pre- and post-intervention.

## Data collection:

Stress levels were assessed using the Perceived Stress Scale (PSS), a validated instrument with scores ranging from 0 (low stress) to 40 (high stress).

## Procedure:

After collecting demographic data and pre-test PSS scores, the experimental group participated in daily 25-minute Tai Chi sessions over 14 days, covering movements like "Rock Forward and Backward" and "Hand Waving the Clouds". Post-intervention PSS scores were then collected from both groups.

## Data analysis:

Paired t-tests compared pre- and post-test stress scores within the experimental group and independent t-tests compared post-test scores between groups. Chi-square tests examined associations between stress reduction and demographic variables.

## Interpretation:

The experimental group experienced a significant reduction in mean stress scores after Tai Chi intervention (p < 0.05), while the control group showed no significant change. Regarding association Between Post-Test Stress Levels and Demographic Variables in the Experimental Group and control group, no significant associations were found between stress reduction and demographic variables in the both group, indicating that Tai Chi's effectiveness was consistent across these demographics.

## Results & Discussion:

**Figure 1:**
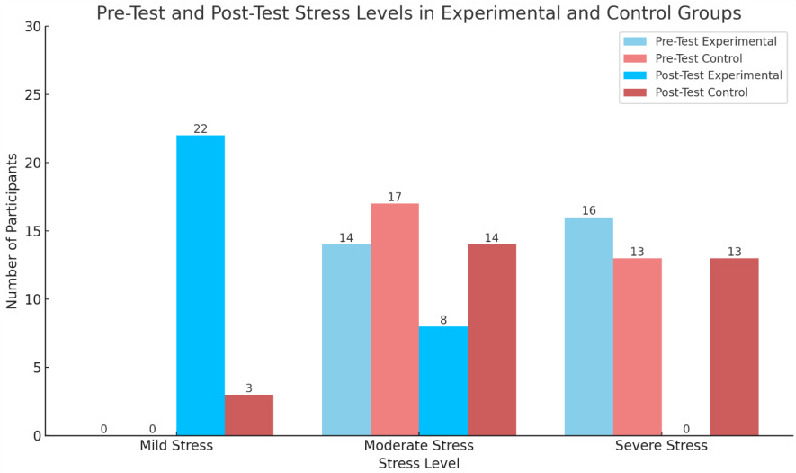
pretest and post-test category in experimental and control group

Table 1 shows the demographic variables of participants in the experimental and control groups. The distribution of age, gender, education, marital status and support system indicates balanced groups, ensuring that differences in stress reduction can be attributed to the Tai Chi intervention. For example, the experimental group had 50% male and 50% female participants, while the control group had a similar gender distribution (46.66% male, 53.33% female). Table 2 presents the mean stress scores pre- and post-intervention for both groups. The experimental group demonstrated a significant reduction in stress scores from 27.2 ± 6.73 to 10.1 ± 5.98 (mean difference = 17.1, t = 10.43, p < 0.05), whereas the control group showed no significant change (mean difference = 1.35, t = 0.78, p > 0.05). These results confirm the effectiveness of Tai Chi in reducing stress levels among older adults. Figure 1 compares the stress levels before and after the intervention in both groups. It highlights the significant reduction in stress among the experimental group, with most participants moving from moderate or severe stress to mild stress. Tai Chi therapy significantly reduces stress among older adults, with participants in the experimental group experiencing a notable shift from moderate or severe stress levels to mild stress after only 14 days of intervention. These findings align with several previous studies highlighting Tai Chi's mental health benefits, though differences in study duration, sample characteristics and intervention intensity present interesting contrasts. The findings of our study are supported by a meta-analysis by yang *et al.* (2021), which reviewed 23 randomized controlled trials and found Tai Chi to significantly reduce stress and anxiety in older adults. However, the studies in yang *et al.*'s review typically had intervention periods of 8 to 12 weeks [10]. 14-day intervention indicates that Tai Chi may have a more immediate impact on stress reduction, suggesting its potential as a rapid-acting stress management tool. Zheng *et al.* (2017) similarly demonstrated the benefits of Tai Chi for mental health, reporting that a 12-week Tai Chi program significantly reduced anxiety and depressive symptoms in older adults with chronic conditions. Their findings, along with our results, underscore Tai Chi's effectiveness across various durations and participant health statuses, further supporting its suitability for community-based stress interventions [11]. Solianik *et al.* (2021) also reported significant reductions in stress following a 10-week Tai Chi intervention among older adults, noting improvements in both physical and mental health outcomes. Their study highlights the dual benefits of Tai Chi for stress relief and physical functioning, aligning with our results [12]. A systemic review by Nan *et al.* (2024) focused on stress reduction in healthy older adults and found that a 6-week Tai Chi intervention significantly reduced perceived stress levels. Although shorter than most Tai Chi programs, this study supports our findings of rapid benefits, suggesting that even brief Tai Chi practices may yield substantial stress relief [13]. Further evidence is provided by Wang *et al.* (2024), who found that a Tai Chi program reduced stress and improved sleep quality in older adults, particularly in those who practiced regularly over an 8-week period. The improved sleep quality observed in Cho *et al.*'s study may be linked to stress reduction, as sleep and stress are often interconnected. This supports our findings and points to the broader holistic benefits of Tai Chi beyond stress alone [14]. Another study by Lee *et al.* (2020) found that while Tai Chi did reduce stress levels in older adults, the effect size was relatively small compared to more intensive physical activities, such as aerobic exercise. Lee *et al.*'s findings contrast with ours and suggest that while Tai Chi is beneficial, it may not be as effective as higher-intensity exercises for certain individuals or under specific conditions. Our findings, supported by several studies, indicate that Tai Chi is a valuable, low-cost and accessible intervention for stress reduction among older adults. Differences observed in Wang *et al.* (2020) suggest that further research is needed to determine the optimal intervention length, session structure and intensity for maximizing Tai Chi's effects on stress. Additionally, future studies could employ objective measures such as cortisol levels to validate self-reported stress reductions and better understand Tai Chi's physiological impacts [15].

## Conclusion:

While our study and supporting literature confirm Tai Chi's effectiveness for stress reduction in older adults, variations across studies highlight the importance of tailoring Tai Chi programs to individual and community needs. Tai Chi emerges as a promising, rapid-acting stress management approach for aging populations, with the potential for wider application in community health initiatives.

## Figures and Tables

**Table 1:** Demographic characteristics of participants

**Demographic variable**	**Category**	**Experimental group (n=30)**	**Control group (n=30)**
Age (years)	60-65	7 (23.33%)	11 (36.66%)
	65-70	11 (36.66%)	11 (36.66%)
	70-75	9 (30%)	5 (16.67%)
	75-80	3 (10%)	3 (10%)
Gender	Male	15 (50%)	14 (46.66%)
	Female	15 (50%)	16 (53.33%)
Education	No formal education	7 (23.33%)	11 (36.66%)
	Elementary school	9 (30%)	13 (43.33%)
	High school	9 (30%)	5 (16.67%)
	Graduate	5 (16.66%)	1 (3.33%)
Marital Status	Married	20 (66.66%)	21 (70%)
	Widow/Widower	8 (26.66%)	7 (23.33%)
Support System	Family members	19 (63.33%)	17 (56.66%)
	Friends	5 (16.66%)	3 (10%)

**Table 2:** Comparison of mean stress scores and hypothesis testing results (pre - and post - test)

**Group**	**Mean pre-test score**	**Mean post-test score**	**Mean difference**	**T-value**	**Significance**
Experimental Group	27.2 ± 6.73	10.1 ± 5.98	17.1	10.43	Significant
Control Group	25.85 ± 6.65	24.5 ± 8.87	1.35	0.78	Not Significant
